# Relation between core strength, core stability, and athletic performance—a mediation analysis approach

**DOI:** 10.3389/fspor.2025.1669023

**Published:** 2025-11-28

**Authors:** Sarah Schulte, Matthias Lukas, Jessica Bopp, Volker Zschorlich, Dirk Büsch

**Affiliations:** 1 Institute of Sport Science, Carl von Ossietzky Universität Oldenburg, Oldenburg, Germany; 2 Institute of Sport Science, Universität Rostock, Rostock, Germany

**Keywords:** ULESS test, trunk, kinematics, diagnostics, lower limb, jumping performance

## Abstract

**Background:**

Core strength and its control in movement, also called core stability, are crucial for athletic performance. However, there is no consensus in the scientific literature regarding the extent of the relation between core strength, core stability, and athletic performance. According to the functional anatomy of the core, it seems that core stability indirectly influences the relation between core strength and athletic performance.

**Objectives:**

This study aimed to examine the relation between core strength, core stability, and athletic performance.

**Methods:**

Forty-one adult sport students were included in a laboratory study. The subjects participated in two testing sessions. Each testing session started with the Unilateral Landing Error Scoring System (ULESS) test. Single-leg drop jumps were performed on force plates to assess jump height as parameter for athletic performance. Drop jumps were recorded from frontal perspective to analyze kinematic data, i.e., lateral pelvic tilt, lateral trunk lean, and frontal knee angles, to evaluate core stability. A testing session involved either isometric core muscle endurance or maximal core strength and core power measurement in four exercises: flexion, extension, lateral flexion right, and lateral flexion left.

**Results:**

A mediation analysis with multiple predictors and multiple mediators was conducted using standardized z-scores of core strength components as predictors, kinematic parameters of core stability as mediators, and jumping performance as the criterion variable. The mediation analysis revealed no statistically significant indirect effects of the mediators on the relation between core strength and jumping performance. Only a small direct effect [β = 0.19, 95% BCa CI (0.10, 0.27), *p* < .001] on the relation between maximal core strength and jumping performance was observed.

**Conclusions:**

The results indicate that, at least in our experimental setup, core stability does not appear to mediate the relation between core strength and jumping performance, but maximal core strength shows a relation to jumping performance. Insufficient force transfer of the hip musculature through the kinetic chain of the drop jump may cause the missing mediating effect of core stability. Consequently, hip strength measurement should be included as an additional predictor or mediator alongside core strength or core stability in the mediation model.

## 1 Introduction

### 1.1 Core strength and core stability

Core strength or core stability has become important in enhancing sports performance (1–9) and skill acquisition (10, 11), improving physical fitness, balance or postural control (1, 12), and reducing the risk of injury to the upper and lower extremities (13–18) and in daily activities (19, 20). Given that the constructs of core strength and core stability have been widely accepted, it is surprising that no clear distinction is made between these constructs (21). In fact, the terms “core strength” and “core stability” are frequently used synonymously, although they originate from different approaches, bear different definitions, and accomplish different functions from an anatomical point of view (21, 22).

In general, the core is anatomically described as a box with the abdominals in the front, the paraspinal and gluteal muscles in the back, the diaphragm as the roof, and the pelvic floor and hip girdle musculature at the bottom (21, 23). The core represents a crucial element within numerous kinetic chains of sporting movements that enable the transfer of mechanical energy to the upper and lower extremities and therefore efficient movement execution (24–27). Functionally, the core musculature can be classified into local, global, and (axial-appendicular) transfer muscle systems (28–30) that generate forces and work synergistically to achieve proximal stability for distal mobility in athletic movements (26, 31). Thus, *core strength* can be defined as the ability of a core muscle or a core muscle group to generate muscular force (32). According to this definition, core strength includes different components, such as the maximal core strength, core endurance, and core power (33). Various types of sports or tasks prioritize the mentioned core strength components to different extents (34).

Neuromuscular control of the core muscles is critical for the precise and coordinated execution of muscle activation, which occurs at the right time, for the correct duration, and with the right combination of forces necessary to control the movement or position of the body. Thus, the body can respond to internal and external as well as expected or unexpected perturbations to ensure static and dynamic core stability (13, 26, 31, 35, 36). Consequently, *core stability* can be defined as a dynamic process that requires optimized core strength (maximal core strength, core endurance, and core power) and neuromuscular control (accurate joint and muscle receptors and neural pathways) that allows efficient integration of external and internal sensory information (13). It seems to be a controlling factor in movement that mediates the direct relation between core strength and athletic performance.

Nevertheless, the distinction between core strength and core stability appears to be ambiguous in the current literature. Accordingly, core muscle endurance tests are predominantly used to represent the construct of core stability or core strength, regardless of their different definitions (21, 34). Few studies have been conducted on the dominant coordinative aspect of core stability in relation to core strength. Small correlations between maximal core strength (e.g., isokinetic) or core muscle endurance (e.g., Biering-Sørensen test, double leg lowering test) and core stability (e.g., sudden loading test, stable and unstable sitting test) were observed (37–39). The findings of these studies revealed that core strength and core stability appear to be distinct components that nevertheless seem to be related to each other. Therefore, different clinical tests should be used to assess the stability and strength of the core. Additionally, it must be considered that specific test performance seems to be associated with the neuromuscular control mechanisms involved in each test (37, 39, 40).

### 1.2 Relation between core strength, core stability, and athletic performance

Adequate core muscle structure and core muscle control are the basis of the kinetic chain for facilitating the transfer of generated forces and moments between the lower and upper extremities in motor tasks of daily life and various sports (25, 26). From a theoretical perspective, core stability appears to mediate the relation between core strength and athletic performance. Thus, the following assumption could be made: An athlete has a high level of core stability when (1) the coordination and (2) the structure of core muscles are adequate so that the produced core strength is sufficient and can be better transferred to the lower and upper extremities, resulting in increased athletic performance. In contrast, an athlete has slight core stability when (1) the coordination and/or (2) the structure of core muscles are inadequate so that the produced core strength is absorbed or limited by neuromuscular control under unstable conditions. Thus, less force is transformed into work of the lower and upper extremities, resulting in decreased athletic performance (41–45).

However, few studies have examined the associations between core strength, core stability, and athletic performance. Prieske et al. (2) listed in their systematic review 15 correlation studies that revealed small relations between core strength and physical performance measures. Bauer et al. (46) reported a small correlation between core muscle endurance and throwing velocity in male handball players. Okada et al. (47) indicated small correlations between core muscle endurance measures and backward medicine ball throw performance. Moreover, several reviews (1–3, 5–9) in recent years regarding the efficacy of core strength or core stability training on athletic performance have reported conflicting results. While Reed et al. (3), Prieske et al. (2), and Saeterbakken et al. (1) revealed small to large effects of core strength or core stability training on physical fitness and moderate effects on sport-specific performance, Dong et al. (6) reported that core training has a large effect on core muscle endurance and general physical fitness parameters of athletes but has a small effect on sport-specific performance. Most correlation and intervention studies include assessment techniques or training programs that focus on the endurance component of the core muscles but do not consider other core strength components, such as maximal core strength and core power, or coordination in core musculature (1, 2, 5, 6, 46, 47). This may be a possible reason why the shared variance between core strength or core stability and athletic performance is only small or why core training has only small effects on sport-specific performance. Consequently, it seems reasonable to consider the core muscular demands (maximal strength, power, and endurance) of a specific sport or task in core strength and core stability assessment and training to improve athletic performance rather than incorporating stimuli that target only the endurance component of the core muscles (6, 34, 38, 48, 49). For example, isometric measurement methods, which assess all core strength components in the same exercise (33, 50, 51), are advisable due to the high standardization of the test conditions. Various studies have suggested that core muscles have a stabilizing function in jumping tasks (e.g., countermovement jumps, drop jumps) (52–55). Moreover, core stability is frequently discussed as a potential risk factor for lower and upper extremity injuries. It is regarded as a crucial element in ensuring the correct positioning of the lower and/or upper extremities (13, 16, 36, 43, 54, 56–59). A comprehensive core strength measurement approach, which considers all components of core strength (33) and a core stability measurement referring to a specific task or sporting movement, is indicated (44).

### 1.3 Aim and hypothesis

The current scientific literature shows conflicting evidence regarding the extent of the relations between core strength, core stability, and athletic performance. Consequently, it has been suggested that core strength or core stability may play a minor role in athletic performance and/or that the assessments used for measuring core strength or core stability may not have been specifically selected for athletic performance requirements. On the basis of the functional anatomy of the core, it is assumed that the core muscles generate core strength, which must be controlled depending on the sporting movement or task to ensure core performance. Consequently, adequate core stability is an intermediary component that leads to increased athletic performance. Following up, we hypothesize that core stability mediates the effect of core strength on athletic performance.

## 2 Materials and methods

The study was conducted in accordance with the Declaration of Helsinki, and the local Ethics Committee of the Carl von Ossietzky Universität Oldenburg, Germany, approved to the protocol (EK/2020/035-08).

### 2.1 Participants

An *a priori* power analysis using the formula by Giraudeau and Mary (60) resulted in a sample size of *N* = 40 participants (see supplementary materials, Sample size calculation). In a randomized controlled study, forty-four adult sports students participated, whereas three subjects were excluded from further analysis for technical reasons. A total of forty-one subjects (*n*_female_ = 20, *n*_male_ = 21, age: 24.0 ± 2.9 years, body height: 178.9 ± 9.9 cm, body mass: 75.2 ± 12.8 kg, body fat: 18.3 ± 6.7%) were included in the final data analysis. The subject had no injuries in the trunk area or the upper or lower extremities at the time of measurement and in the previous twelve months, nor were they receiving treatments. The participants represent a diverse range of athletic backgrounds (*N* = 26 different sports; e.g., handball, soccer, gymnastics, volleyball). A limited number of subjects (*n* = 15) engaged in targeted core strength training (49.7 min ± 51.1 min) every week. The determination of leg dominance (*n*_right_ = 20, *n*_left_ = 21) was based on the question of which leg is used for take-off in the long jump (dominant leg = jumping leg, nondominant leg = swinging leg).

### 2.2 Procedures

**Figure 1:**
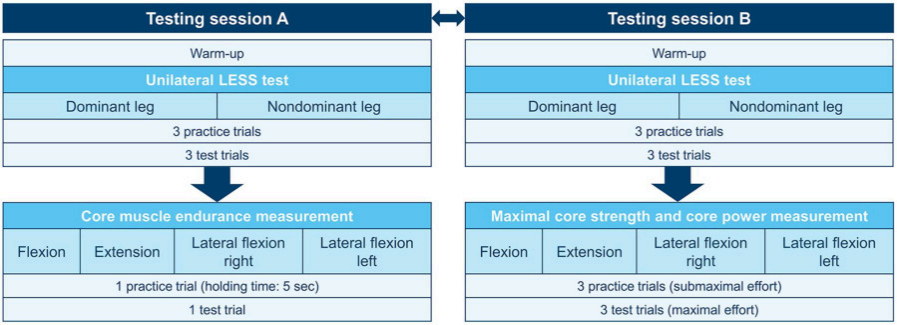
Study design.

All subjects participated in two testing sessions (see Figure 1), each lasting 90 minutes, in a controlled laboratory environment. Before the commencement of the experimental procedure, the subjects were duly informed about the methodology and provided written consent to participate in the study. In the first of the two testing sessions, the subjects completed a questionnaire that included personal information, sports background, and injury history. The participants were similarly recovered at the beginning of both testing sessions. Anthropometric measurements (body mass, body height, and body fat) were performed via the Inbody270 (Inbody Co., Seoul, Korea) and a stadiometer (Seca GmbH & Co. KG, Germany). Both testing sessions began with a five-minute warm-up to activate the entire musculature of the body, with particular attention to the core. The Unilateral Landing Error Scoring System test (ULESS test) (61) was subsequently conducted to evaluate the core stability and athletic performance data. The order of the starting leg (dominant or nondominant) in the ULESS test was randomized but consistent across both testing sessions. Following the core stability assessment, one of the two testing sessions was performed: Testing session A included the core muscle endurance measurement, and testing session B included the measurement of maximal core strength and core power. The order of the testing sessions was randomized. There were at least seven days between the two testing sessions.

### 2.3 Instruments

#### 2.3.1 Core stability and athletic performance measurement

**Figure 2:**
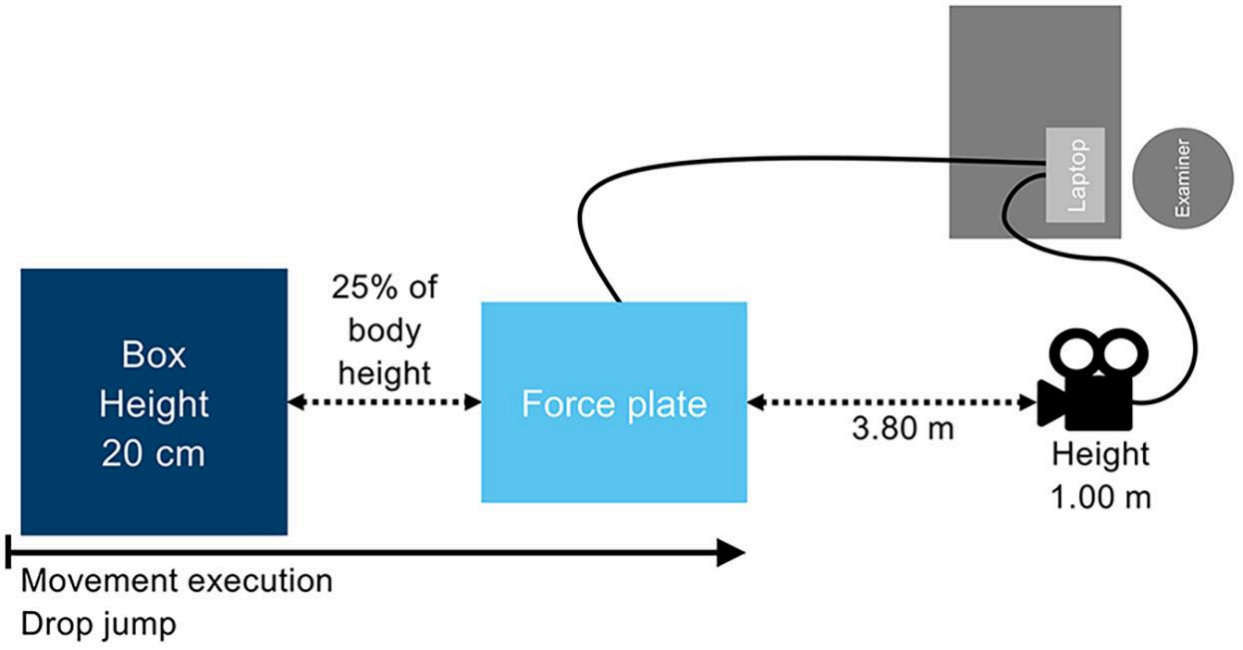
Experimental set-up.

The subjects were asked to perform the ULESS test, which is regarded as a reliable tool for the qualitative assessment of movement kinematics (61, 62). The ULESS test was used to assess both *core stability* and *athletic performance* in a sport-specific manner. Single-leg jumps are common in various sports. On the one hand, they are important for athletic performance, and on the other hand, they represent a central injury situation that is considered in injury prevention (15). The subjects stood on a 20 cm high box with both feet pointing forward, approximately at shoulder width. The box was placed at a distance of 25% of the individual's body height from a force plate (Kistler AG, type 9260AA6, frequency 400 Hz, Winterthur, Switzerland). The experimental set-up of the ULESS test is shown in Figure 2.

The subjects were instructed to perform a horizontal drop from the box toward the force plate with a bilateral take-off, landing unilaterally on the force plate, and jumping with the same leg vertically and as high as possible immediately after ground contact. Following the completion of the vertical jump, a bilateral landing was permitted on the force plate. The arms had to be placed on the hips during the whole task (61). To familiarize themselves with the task, the subjects had three practice trials for each leg. Afterwards, three test trials were conducted for each leg, with a 30-second rest interval between trials. A two-minute rest interval was observed during the transition to the other leg. Trials were considered successful when the subjects (1) performed symmetrical bilateral take-off from the box, (2) landed completely and unilaterally on the force plate, (3) and performed a vertical, unilateral jump in fluid motion, and landed with both feet on the force plate. The performances were recorded in the frontal plane using a high-speed camera (VCXU-50C, 100 Hz sampling frequency, Baumer Holding AG, Frauenfeld, Switzerland). The frontal camera was positioned at a distance of 3.80 m from the force plate and at a height of 1 m. The 2D video data were processed by movement analysis software TEMPLO® (Version 2022.1, Contemplas GmbH, Kempten, Germany).

#### 2.3.2 Core stability analysis

**Figure 3:**
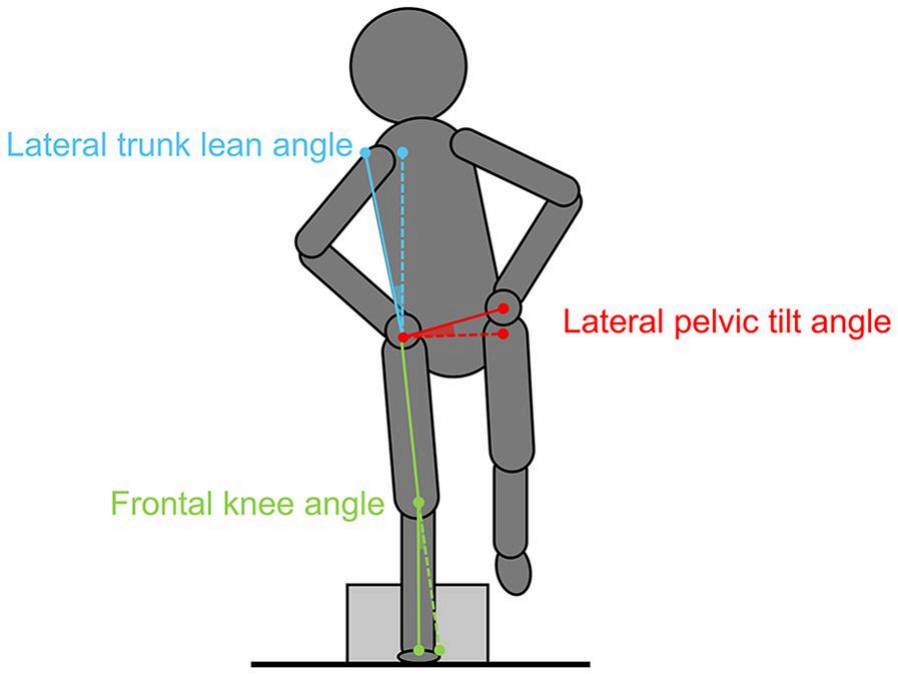
Kinematic parameters for core stability in single-leg drop jump.

Different studies have shown a relation between various biomechanical variables of the core kinematics and lower limb kinematics during jumping tasks (14, 15, 57, 58). It has been proposed that the control of the vertical axis of the upper body, the knee, and the horizontal axis of the pelvis in unilateral movements should be key parameters for assessing core stability (see Figure 3). Therefore, the lateral trunk lean, lateral pelvic tilt, and frontal knee angles in the first ground contact of the drop jump at the moment of maximal knee flexion of the jumping leg (63–65) were determined using movement analysis software (Kinovea, version 0.9.5) (61). The frame at peak knee flexion was visually identified for 2D video analysis (63). The lateral trunk lean angle (blue angle in Figure 3) is formed by the lateral shoulder joint center and the anterior superior iliac spine (ASIS) of the jumping leg side. A line connects these landmarks, and the angle of the frontal plane is determined (66). The difference between the baseline lateral trunk lean angle in the standing position before jumping and the lateral trunk angle at the time of the first ground contact was calculated. A negative value characterized a lateral trunk leaning toward the jumping leg, and a positive value characterized a lateral trunk leaning toward the swinging leg. For the frontal knee angle (green angle in Figure 3), a line was drawn from the ASIS to the center of the patella and from the patella to the center of the ankle joint of the jumping leg. The angle of the frontal plane was calculated (64). Positive values indicate a valgus knee position, and negative values indicate a varus knee position. The lateral pelvic tilt angle (red angle in Figure 3) was determined through the height difference between the left and right ASIS. The ASISs were connected with a horizontal line starting at the ASIS of the jumping leg. The angle of the horizontal plane was defined (63). A positive value represented a contralateral pelvis rise, whereas a negative value represented a contralateral pelvis drop. Absolute values of the core stability variables were determined under the assumption that core stability decreases with increasing distance from zero degrees in the vertical axis of the spine (lateral trunk lean angle) and knee (frontal knee angle) and the horizontal axis of the pelvis (lateral pelvic tilt angle), regardless of the direction.

#### 2.3.3 Athletic performance analysis

The jump height and ground contact time at the first ground contact of the drop jump in the ULESS test were automatically calculated by movement analysis software TEMPLO® (Version 2022.1, Contemplas GmbH, Kempten, Germany). Therefore, the ground reaction force signals of the force plate for the drop jumps were synchronized and automatically transformed into jump height and ground contact time values. Jump height values were used for the statistical analysis, while the ground contact time was taken to control the stretch-shortening cycle of the participants.

#### 2.3.4 Core strength measurement

All strength components (endurance, maximal strength, and power) of the core muscles were measured in a lying position via the abdominal flexion test (anterior abdominal muscles), the Biering-Sørensen test (back muscles), and the lateral flexion test (oblique muscles) (33, 50, 51). The order of tests remained consistent for all participants following a prescribed sequence for technical reasons: abdominal flexion, back extension, lateral flexion right, and lateral flexion left (33). The positions in each test were standardized and controlled manually using a goniometer. The parameters of holding time, maximal voluntary isometric contraction (MVC), and peak rate of force development (pRFD) were measured in each of the four exercises. The instruction provided to the participants within the holding time measurement was to maintain the different test positions for as long as possible. A stopwatch was used by the examiner to record the holding time in seconds. The participants performed one familiarization trial for a maximum duration of five seconds, followed by a test trial in each test position (flexion, extension, lateral flexion right, and lateral flexion left). Five minutes of rest between the test positions was permitted. In MVC and pRFD measurement, the participants were instructed to pull from a light preload as hard and fast as possible on the force sensor for a duration of five seconds (67, 68). Three practice trials with submaximal effort were permitted for each test position, followed by three test trials with maximal effort. The participants rested one minute between each test trial in a single test position and two minutes during transition between positions. The processing of the force-time curves was undertaken using MuscleLab software (Version 10.200.90.5097, Ergotest Innovation AS, Stathelle, Norway) with a sampling rate of 1 kHz (68). The MVC values were extracted as a 20-millisecond moving average from the raw data for each test trial. Moreover, the pRFD values were also determined by a 20-millisecond moving average in force-time curves. The mean MVC and pRFD values of the three test trials in each position were used for further analysis. A comprehensive description and further details of the test setup of the core strength component measurements have been provided by Schulte et al. (33).

### 2.4 Statistical analysis

The data were statistically analyzed using JASP (version 0.18.3, University of Amsterdam, Netherlands). First, the data were checked with the Shapiro–Wilk test for normal distribution and further examined for skewness, kurtosis, and unimodality (69). The means and standard deviations of core strength variables, core stability variables, and drop jump heights were calculated for each testing session and pooled for the dominant and nondominant legs. The intraclass correlation coefficients (*ICCs*) for the core strength variables, core stability variables, and the drop jump height were calculated to determine test-retest reliability (2-way mixed effects, absolute agreement, multiple raters or measurements). The test-retest reliability of core stability variables and drop jump height was determined by the three ULESS test trials in the first testing session. Test-retest reliability of MVC and pRFD variables was determined using the three test trials for maximal core strength and core power measurement. The *ICC* values were interpreted according to Koo and Li (70) as poor (< 0.50), moderate (0.50–0.75), good (0.75–0.90), or excellent (> 0.90). Moreover, the standard error measurement (*SEM*) was computed as $SEM=SD\;x\;\sqrt{\;1\;\text{-}\;ICC}$ (71), and the coefficient of variation (*CV*) was calculated as $CV=\frac{SD}{M}*100$ to evaluate the degree of variation between measurements. All *CV* values < 10% can be considered acceptable according to Cormack et al. (72). The interrater and intrarater reliability (2-way mixed effects, consistency of multiple raters or measurements and 2-way mixed effects, absolute agreement, multiple raters or measurements, respectively) of core stability variables (lateral trunk lean, frontal knee, and lateral pelvic tilt angles) were quantified using the *ICCs* following the recommendations of Koo and Li (70). Two raters screened and rated a selection of 82 recordings (one random trial of each subject for the dominant and nondominant legs). The values of the core stability variables of the two raters were summarized for the dominant and nondominant legs and compared to compute interrater reliability (.83 ≤ *ICC* ≥ .99). Moreover, one rater re-rated the same 82 recordings a second time, at least one week apart, to calculate intrarater reliability (.88 ≤ *ICC* ≥ .99) combined for both the dominant and nondominant legs (see supplementary material Table 1). Inter- and intrarater reliability separated for both the dominant and nondominant legs are represented in Supplementary material Table 2.

**Figure 4:**
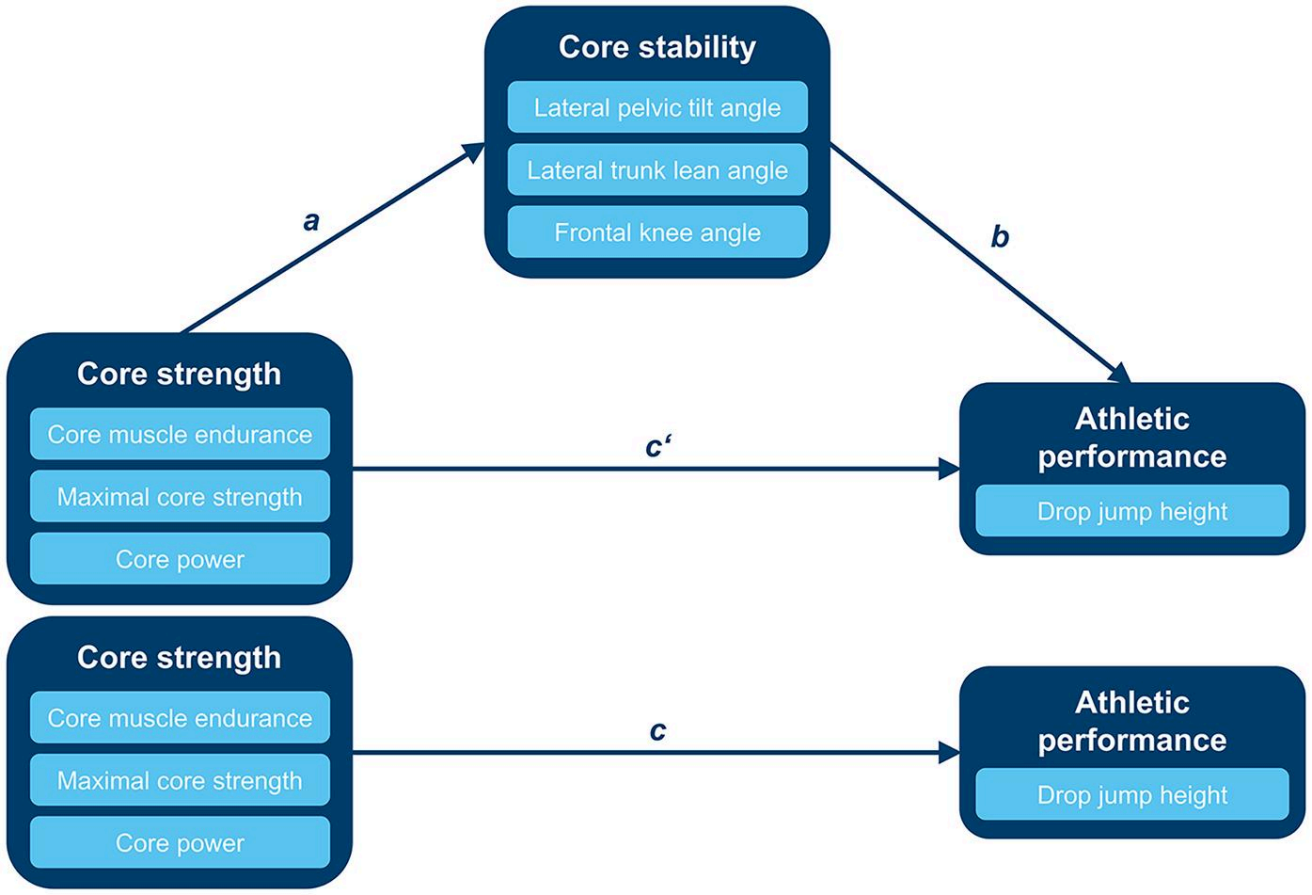
Mediation model of the relations between core strength, core stability, and athletic performance (*ab,* indirect effect; *c’*, direct effect; *c*, total effect).

The means and standard deviations of core stability and jumping performance variables were further summarized for both testing sessions and both the dominant and nondominant legs. All variables were *z*-standardized to indicate the relations between core strength variables, core stability variables, and drop jump height in the mediation analysis. Before running the mediation analysis, a principal component analysis (PCA) was used to reduce the dimensionality of the core strength data. The *z*-standardized values of holding time, MVC, and pRFD variables of the four exercises (flexion, extension, lateral flexion right, and lateral flexion left) in core strength measurement were included in the PCA. Through the orthogonally varimax rotation, the variables were assigned to the component on which they loaded higher than on the other components. The PCA extracted three principal components out of twelve different core strength variables, which explained 73.4% of variance. MVC variables loaded high on one component, pRFD variables loaded high on one component, and holding time variables loaded high on one component. Based on the results of the PCA, the holding time, MVC, and pRFD values in the flexion, extension, lateral flexion right, and lateral flexion left exercises of the core strength measurement were summed into three components: core muscle endurance, maximal core strength, and core power. According to the theoretical hypothesis, core strength variables (maximal core strength, core power, and core muscle endurance) were considered as predictor variables, core stability parameters (lateral trunk lean, frontal knee, and lateral pelvic tilt angles) demonstrated the mediator variables, and the athletic performance variable drop jump height was characterized as the criterion variable in the simplified mediation model (see Figure 4).

Statistical mediation analysis was performed to expand the understanding of how core strength affects athletic performance through the indirect effect of core stability. If the observed relation between the predictor and criterion variables becomes weaker after the inclusion of an objectively measured mediator variable, partial mediation will occur. If the size of the association between the predictor variable and the criterion variable was not significant and the confidence interval of the beta-coefficient included zero after the inclusion of an objectively measured mediator variable, complete mediation would be observed (73). Multiple predictors (core muscle endurance, maximal core strength, and core power) and multiple mediators (lateral pelvic tilt, lateral trunk lean, and frontal knee angles) were included in the mediation analysis which was performed with JASP (version 0.18.3, University of Amsterdam, Netherlands) based on an R package for structural equation modeling of Rosseel (74). The mediation hypothesis test was estimated using a confidence interval by the bootstrapping method with bias-correction and acceleration (95% BCa, 5000 resamplings) (73). The bootstrapping method is a non-parametric approach by Preacher and Hayes (75) to estimate the effect size and hypothesis testing that does not rely on the assumption about the distributions of the variables and applies to small sample sizes. The method is accomplished by creating new samples from the original data, sampling with replacement is conducted, and the indirect effect is computed for each sample (75). The effect size of *R^2^* was determined to assess the explained variance accounted for in the mediation model. Thus, both the total effect and component paths in the mediation model can be evaluated (76). The effect size of *R^2^* was interpreted according to Cohen (77) as small (*R^2^* = .02), medium (*R^2^* = .13), and large (*R^2^* = .26).

## 3 Results

The assumption of normal distribution for all variables can be maintained. Descriptive data, test-retest reliability (*ICC*), *SEM*, and *CV* of the core stability, core strength, and athletic performance variables are presented in Table 1. Test-retest reliability was considered moderate to good (.72 ≤ *ICC* ≥ .88) for core stability variables, good to excellent (.82 ≤ *ICC* ≥ .99) for core strength variables, and excellent (*ICC* = .96) for the athletic performance variable. The *SEM*s of 0.8–1.5°, 11.1–17.3 N, 150.8–335.2 N/s, and 0.01 m were observed for core stability, MVC, pRFD, and athletic performance variables, respectively. The core stability, MVC, and pRFD variables displayed *CV* ranges of 19.3–24.1%, 5.5–11.8%, and 24.0–29.8%, respectively. The drop jump height shows a *CV* of 8.2%. Test-retest reliability separated for both the dominant and nondominant legs is represented in Supplementary material Table 3.

**Table 1:** Descriptive statistics and test-retest reliability of the core stability, core strength, and athletic performance variables.

	*M*	*SD*	*ICC*	95% CI	*SEM*	*CV* [%]
Core stability variables^a^						
Lateral pelvic tilt angle [°]	5.1	1.4	.72	.53, .84	0.8	24.1
Lateral trunk lean angle [°]	10.1	2.9	.84	.74, .91	1.1	19.3
Frontal knee angle [°]	9.9	4.3	.88	.80, .93	1.5	28.3
Core strength variables						
*Holding time [s]*						
Flexion	223.4	138.5	-^b^	-^b^	-^b^	-^b^
Extension	161.3	49.4	-^b^	-^b^	-^b^	-^b^
Lateral flexion right	65.5	28.6	-^b^	-^b^	-^b^	-^b^
Lateral flexion left	73.3	28.9	-^b^	-^b^	-^b^	-^b^
*MVC [N]*						
Flexion	294.6	102.1	.99	.97, .99	12.5	5.5
Extension	316.7	85.4	.96	.93, .98	17.3	8.5
Lateral flexion right	150.5	71.4	.98	.96, .99	11.1	11.6
Lateral flexion left	162.5	72.4	.96	.93, .98	15.0	11.8
*pRFD [N/s]*						
Flexion	1,895.9	939.1	.88	.78, .93	332.0	29.2
Extension	1,600.9	805.9	.83	.71, .90	335.2	29.8
Lateral flexion right	750.7	356.4	.82	.68, .91	150.8	24.0
Lateral flexion left	753.5	453.7	.88	.80, .94	155.9	24.8
Athletic performance variable						
Drop jump height [m]	0.121	0.036	.96	.94, .98	0.010	8.2

CI, confidence interval; *CV*, coefficient of variation; *ICC*, intraclass correlation coefficient; *M*, mean value; MVC, maximal voluntary isometric contraction; pRFD, peak rate of force development; *SD*, standard deviation; *SEM*, standard error of measurement.

a Absolute values of core stability variables.

b For methodological reasons, only one trial was recorded.

The total (*c*), direct (*c’*), and indirect effects (*ab*) of the mediation analysis are presented in Table 2. The relations between core strength variables (core muscle endurance, maximal core strength, and core power), core stability variables (lateral pelvic tilt angle, lateral trunk lean angle, and frontal knee angle), and drop jump height showed no statistically significant indirect effects (see Table 2). The direct effect estimated by the mediation model revealed a statistically significant positive relation between maximal core strength and drop jump height [*c’* = 0.19, 95% BCa CI (0.10, 0.27), *p* < .001]. No statistically significant direct effects were observed between core muscle endurance and drop jump height, nor between core power and drop jump height. The total effect of the mediation analysis was statistically significant for the relation between maximal core strength and drop jump height [*c* = 0.21, 95% BCa CI (0.13, 0.28), *p* < .001]. There were no statistically significant total effects between core muscle endurance and drop jump height, and between core power and drop jump height. Nevertheless, specific path coefficients of the mediation model were statistically significant (see supplementary material Table 4). The core strength variables together explained. 10 ≤ *R^2^* ≥ .34 of variance in the core stability variables (*a* paths) and *R^2^* = .52 of variance in jumping height (*c* paths). The core stability variables together explained *R^2^* = .19 of variance in jumping height (*b* paths). The core strength and core stability variables together explained a variance of *R^2^* = .57 in the drop jump height (*ab* paths). In addition, separate mediation analyses were performed for both the dominant and nondominant legs, which yielded similar results (see supplementary material Tables 5, 6).

**Table 2:** Total, direct, and indirect effects of the mediation analysis.

Effect	β	*SE*	95% BCa CI	*z*	*p*
Total effect (*c*)					
CME → DJH	−0.007	0.04	−0.08, 0.06	−0.19	.850
MCS → DJH	0.21	0.04	0.13, 0.28	5.11	<.001
CP → DJH	0.008	0.04	−0.07, 0.10	0.20	.839
Direct effect (*c’*)					
CME → DJH	−0.03	0.05	−0.14, 0.06	−0.60	.549
MCS → DJH	0.19	0.04	0.10, 0.27	4.57	<.001
CP → DJH	0.03	0.05	−0.06, 0.15	0.60	.549
Indirect effect (*ab*)					
CME → LPT → DJH	0.002	0.01	−0.02, 0.04	0.20	.845
CME → LTL → DJH	0.007	0.01	−0.01, 0.05	0.80	.422
CME → FKA → DJH	0.01	0.02	−0.04, 0.06	0.65	.514
MCS → LPT → DJH	0.03	0.02	−0.004, 0.08	1.45	.146
MCS → LTL → DJH	< −0.001	0.01	−0.03, 0.02	−0.03	.977
MCS → FKA → DJH	−0.01	0.02	−0.07, 0.03	−0.65	.516
CP → LPT → DJH	−0.03	0.02	−0.09, 0.01	−1.57	.117
CP → LTL → DJH	0.01	0.01	−0.01, 0.08	0.96	.340
CP → FKA → DJH	−0.01	0.01	−0.05, 0.01	−0.58	.564

β, beta-coefficient; BCa CI, bias-corrected and accelerated bootstrap confidence interval; CME, core muscle endurance; CP, core power; DJH, drop jump height; FKA, frontal knee angle; LPT, lateral pelvic tilt angle; LTL, lateral trunk lean angle; MCS, maximal core strength; *p*, p-value; *SE*, standard error; *z*, z-value.

## 4 Discussion

This study aimed to examine the relation between core strength, core stability, and athletic performance. The hypothesis was that core stability mediates the relation between core strength and athletic performance (specifically: jumping performance). The main findings indicate that core stability (none of the selected parameters) does not seem to mediate the relation between core strength and athletic performance. In contrast, core strength, particularly maximal core strength, appeared to be directly related to athletic performance with a small effect size (β = 0.19), whereas core muscle endurance and core power did not. Thus, the mediating hypothesis that core stability mediates the relation between core strength and athletic performance could not be verified. According to Zhao et al. (78), these results can be interpreted as direct-only non-mediation.

### 4.1 Causes of non-mediating core stability

The absence of mediation could result from an insufficient transfer of force between the core and lower extremities in the kinetic chain (26). Functionally, in addition to the local and global system of the core, the hip musculature (e.g., hip flexors, hip extensors, hip abductors, and hip adductors) is classified as an axial-appendicular musculature that connects the lower extremities to the pelvic girdle of the core and transfers forces through the kinetic chain between the core and the lower extremities during movement (29, 30). Studies investigating the correlations between hip muscle strength and control of the core and lower extremities in single-leg activities (e.g., jumping, step-down task, squat, bridge) have indicated that weak hip abductors may alter muscle activation to control and stabilize the core and lower extremities (79–83). The gluteal muscles (e.g., the gluteus maximus and gluteus medius) appear to play an important role in the frontal plane stability of the pelvis, trunk, and lower extremities during jumping tasks (80, 82). The gluteus maximus is attached to the pelvis and lumbar spine via the thoracolumbar fascia (84). In our study, the hip musculature may have been unable to adequately support the mass of the body during the first ground contact of the drop jump, resulting in compensatory movement of the trunk and/or lower extremities to maintain stability. The hip muscles may not have been able to transfer forces effectively between the lower extremities and core, meaning that the mediating influence of core stability on the relation between core strength and athletic performance of the lower extremities could not be observed. Resende et al. (79) reported that core strength and hip strength predict part of the variability in core stability. Hip strength could be included in an analysis (1) alongside core strength as an additional predictor or (2) alongside core stability as an additional mediator/moderator because of its ability to transfer between the lower extremities and the core. Although no mediating effect of core stability could be indicated, the total mediation model already shows a large effect size (*R^2^* = .57). Extending the mediation model with an additional component may improve the variance accounted for by the component paths (*a* paths) in the mediation analysis. Further studies could indicate a potentially mediating effect of core stability on the relation of core and hip strength with athletic performance of lower extremities in drop jumps. Alternatively, the potentially mediating effect of core stability and hip strength on the relation between core strength and athletic performance of the lower extremities could be revealed. Despite the absence of a mediating effect of core stability on the relation between core strength and athletic performance in the present study, a distinction was made between the theoretical constructs of core strength and core stability, considering their divergent definitions, in contrast to most previous studies. In accordance with a small number of studies (37, 40), different measurement methods have been employed to quantify (1) isolated core strength, with a focus on the force-generating task of distinct core muscle groups; and (2) core stability, with a focus on the coordination aspect of core muscles during athletic movement. A more thorough examination of the relation between core strength and core stability revealed very small correlations (see supplementary material Table 2). Consistent with our study, similar results have been reported by other studies (37, 40, 85, 86).

In our study, both core stability and athletic performance were determined with drop jump movements to achieve a high degree of representativeness and a potential correlation between core stability and athletic performance following a head-to-toe approach (44). From the perspective of the principle of the kinetic chain (26), poorer athletic performance is expected when the axial stability of the spine, pelvis, and knee in the frontal plane varies in the first ground contact of the drop jump. Nevertheless, in the current study, trivial to small correlations were indicated between core stability and jumping performance (see supplementary material Table 4). Our study partially revealed a weak relation between core stability and athletic performance. In the current literature, there is a paucity of evidence regarding the relation between core stability and athletic performance (87, 88). More evidence is necessary for a comprehensive understanding of how core stability works in the different planes of movement and how it's related to athletic performance (37).

### 4.2 Relation between core strength and jumping performance

In contrast to most studies, which focus on core muscle endurance in relation with athletic performance (2, 6, 21, 34), our study used a systematic and comprehensive approach to evaluate maximal core strength, core power, and core muscle endurance under highly standardized conditions (33). The results of the present study indicate that maximal core strength is related to jumping performance, whereas core muscle endurance and core power are not. In relation to the jumping performance of the drop jump, it appears that maximal core strength contributes more than core power and core muscle endurance. The single-leg drop jump is a dynamic movement with a high physical load during the initial ground contact. According to the general classification of strength (89), it seems reasonable that maximal core strength, as a basic dimension, was related to jumping performance rather than core muscle endurance and core power. Similar to our study, Prieske et al. (52) revealed positive correlations between maximal core strength of the extensor muscles and drop jump height under stable (*r* = 0.64) and unstable conditions (*r* = 0.66). Other studies investigating the relation between core power as well as core muscle endurance and jumping performance, have yielded inconclusive results (41, 53, 90–94).

### 4.3 Limitations and future directions

Readers should note the following limitations of this study. The kind of muscle action involved in isometric core strength measurements and during drop jumps may not be similar. Thus, dynamic core strength measurements, in addition to isometric core strength measurements, could be considered in further studies (95). The results of this study reveal a relatively high coefficient of variation for certain core power and core stability variables without any systematic biases (e.g., learning or fatigue effects). In future studies, further methodological measures should be taken to improve data quality. The sex and training background of the participants may be potential moderators in the mediation model, which can influence the results. Further studies with larger sample sizes should consider potential sex differences and differences in training background in core strength (96) and biomechanical measurements (e.g., frontal knee angle) (97). Moreover, the current study first of all provides results on core stability in the frontal plane of movement during a drop jump. Further studies should consider biomechanical measurements in different planes of movement to evaluate core stability during movements more precisely and comprehensively in relation to athletic performance.

## 5 Conclusion

The present study revealed that core stability, with the parameters selected in our study, does not seem to mediate the relation between core strength and jumping performance, but a direct relation between core strength, particularly maximal core strength, and jumping performance was observed. Our current approach suggests that the hip muscles do not adequately transfer forces between the core and lower extremities during the initial ground contact of the drop jump, thereby altering the motor control mechanisms of the core and lower extremities. Future studies should measure hip strength as an additional mediator alongside core stability or as an additional predictor alongside core strength to identify a mediating effect of core stability on the relation of core strength with athletic performance of the lower extremities. A more profound understanding of the interactions between core strength, hip strength, core stability, and athletic performance will facilitate the development of assessments and training that are tailored to the specific demands of different tasks or sports by sport scientists and practitioners.

## Data Availability

The original contributions presented in the study are included in the article/Supplementary Material, further inquiries can be directed to the corresponding author.
