# Supplementary material for: Relation between core strength, core stability, and athletic performance—a mediation analysis approach
Source: Front Sports Act Living. 2025 Nov 28;7:1669023. doi: 10.3389/fspor.2025.1669023 (PMC12698640; doi:10.3389/fspor.2025.1669023)
Supplement: Supplementary file 2 [file Table2.docx]

Supplementary Material

Sample size calculation

The sample size was determined using the formula by Giraudeau and Mary (2001):

$\text{n}\text{ =} \frac{\text{8}{\text{z}_{\text{1-}\text{∝}/\text{2}}}^{\text{2}} {\text{(1-}\text{ICC}\text{)}}^{\text{2}} {\text{[1+(}\text{m-}\text{1) }\text{ICC}\text{]}}^{\text{2}}}{\text{m}\left( \text{m}\text{-1} \right){\text{ }\text{w}}^{\text{2}}} (\text{z}_{\text{1-}\text{∝}/\text{2}}\text{ = 1.96; }\text{ICC}\text{ = 0.9; }\text{m }\text{= 3; }\text{w }\text{= 0.1)}$ (1)

$\text{z}_{\text{1-}\text{∝}/\text{2}}\text{ }$= quantile of standard normal distribution, *m* = number of measurements, *ICC* = intraclass correlation coefficient, *w* = total width of the 100(1-α) % confidence interval

Table 1: Intrarater and interrater reliability of core stability variables

|  | **Intrarater reliability (*N* = 41)** | | **Interrater reliability** **(*N* = 41)** | |
| --- | --- | --- | --- | --- |
| **Variable** | *ICC* | 95% CI | *ICC* | 95% CI |
| Lateral pelvic tilt angle | .88 | .77, .93 | .83 | .67, .91 |
| Lateral trunk lean angle | .95 | .90, .97 | .86 | .73, .92 |
| Frontal knee angle | .99 | .99, >.99 | .99 | .98, >.99 |
| CI, confidence interval; *ICC*, intraclass correlation coefficient | | | | |

Table 2: Intrarater and interrater reliability of core stability variables separated for both the dominant and nondominant legs

|  | **Dominant leg (*N* = 41)** | | | | **Nondominant leg (*N* =41)** | | | |
| --- | --- | --- | --- | --- | --- | --- | --- | --- |
|  | Intrarater reliability | | Interrater reliability | | Intrarater reliability | | Interrater reliability | |
| **Variable** | *ICC* | 95% CI | *ICC* | 95% CI | *ICC* | 95% CI | *ICC* | 95% CI |
| Lateral pelvic tilt angle | .92 | .85, .96 | .86 | .74, .93 | .84 | .69, .91 | .83 | .68, .91 |
| Lateral trunk lean angle | .91 | .83, .95 | .87 | .76, .93 | .95 | .91, .97 | .90 | .81, .95 |
| Frontal knee angle | .99 | .98, >.99 | .99 | .98, >.99 | .99 | .98, >.99 | .99 | .98, .99 |
| CI, confidence interval; *ICC*, interclass correlation coefficient | | | | | | | | |

Table 3: Test-retest reliability of core stability and athletic performance variables separated for both the dominant and nondominant legs

|  | **Testing session 1** | | | | **Testing session 2** | | | |
| --- | --- | --- | --- | --- | --- | --- | --- | --- |
|  | Dominant leg | | Nondominant leg | | Dominant leg | | Nondominant leg | |
| **Variable** | *ICC* | 95% CI | *ICC* | 95% CI | *ICC* | 95% CI | *ICC* | 95% CI |
| Lateral pelvic tilt angle | .85 | .75, .91 | .77 | .62, .87 | .73 | .56, .85 | .85 | .74, .91 |
| Lateral trunk lean angle | .84 | .74, .91 | .77 | .60, .87 | .81 | .69, .89 | .87 | .78, .93 |
| Frontal knee angle | .92 | .87, .96 | .92 | .87, .96 | .92 | .86, .95 | .96 | .94, .98 |
| Jump height | .95 | .92, .97 | .92 | .87, .96 | .96 | .94, .98 | .94 | .90, .97 |
| CI, confidence interval; *ICC*, interclass correlation coefficient | | | | | | | | |

Table 4: Path coefficients of mediation analysis

| **Path coefficients** | **β** | ***SE*** | **95 % BCa CI** | ***z*** | ***p*** |
| --- | --- | --- | --- | --- | --- |
| LPT → DJH | -0.23 | 0.13 | -0.44, 0.09 | -1.75 | .081 |
| LTL → DJH | 0.15 | 0.15 | -0.17, 0.41 | 1.01 | .311 |
| FKA → DJH | 0.09 | 0.16 | -0.23, 0.39 | 0.55 | .580 |
| CME → DJH | -0.03 | 0.05 | -0.14, 0.06 | -0.60 | .549 |
| MCS → DJH | 0.19 | 0.04 | 0.10, 0.27 | 4.57 | < .001 |
| CP → DJH | 0.03 | 0.05 | -0.06, 0.15 | 0.60 | .549 |
| CME → LPT | -0.01 | 0.05 | -0.11, 0.08 | -0.20 | .841 |
| MCS → LPT | -0.11 | 0.06 | -0.23; -0.01 | -1.96 | .050 |
| CP → LPT | 0.13 | 0.05 | 0.03, 0.23 | 2.47 | .013 |
| CME → LTL | 0.05 | 0.06 | -0.06, 0.16 | 0.91 | .364 |
| MCS → LTL | -0.002 | 0.05 | -0.11, 0.11 | -0.03 | .997 |
| CP → LTL | 0.07 | 0.06 | -0.04, 0.21 | 1.20 | .229 |
| CME → FKA | 0.14 | 0.04 | 0.08, 0.21 | 3.91 | < .001 |
| MCS → FKA | -0.14 | 0.05 | -0.24, -0.05 | -2.86 | .004 |
| CP → FKA | -0.05 | 0.05 | -0.15, 0.05 | -1.05 | .296 |
| β, beta-coefficient; BCa CI, bias corrected and accelerated bootstrap confidence interval; CME, core muscle endurance; CP, core power; DJH, drop jump height; FKA, frontal knee angle; LPT, lateral pelvic tilt angle; LTL, lateral trunk lean angle; MCS, maximal core strength; *p*, p-value; *SE*, standard error; *z*, z-value | | | | | |

Table 5: Total, direct and indirect effects of mediation analyses separated for both the dominant and nondominant legs

|  | **Dominant leg** | | | | | **Non-dominant leg** | | | | |
| --- | --- | --- | --- | --- | --- | --- | --- | --- | --- | --- |
| **Effect** | β | *SE* | 95% BCa CI | *z* | *p* | β | *SE* | 95% BCa CI | *z* | *p* |
| **Total effect (*c*)** |  |  |  |  |  |  |  |  |  |  |
| CME → DJH | -0.02 | 0.04 | -0.09, 0.05 | -0.40 | .690 | 0.003 | 0.04 | -0.08, 0.07 | 0.08 | .935 |
| MCS → DJH | 0.20 | 0.04 | 0.12, 0.27 | 5.02 | < .001 | 0.20 | 0.04 | 0.13, 0.29 | 4.78 | < .001 |
| CP → DJH | 0.02 | 0.04 | -0.05, 0.10 | 0.49 | .624 | -0.007 | 0.04 | -0.09, 0.09 | -0.16 | .872 |
| **Direct effect (*c’*)** |  |  |  |  |  |  |  |  |  |  |
| CME → DJH | -0.05 | 0.04 | -0.13, 0.02 | -1.43 | 0.154 | 0.008 | 0.04 | -0.09, 0.08 | 0.19 | .852 |
| MCS → DJH | 0.21 | 0.03 | 0.15, 0.28 | 6.44 | < .001 | 0.14 | 0.05 | 0.03, 0.23 | 2.87 | .004 |
| CP → DJH | 0.02 | 0.04 | -0.05, 0.10 | 0.46 | 0.644 | -0.009 | 0.05 | -0.09, 0.11 | -0.18 | .857 |
| **Indirect effect (*ab*)** |  |  |  |  |  |  |  |  |  |  |
| CME → LPT → DJH | < -0.001 | 0.002 | -0.03, 0.01 | -0.15 | .880 | 0.006 | 0.01 | -0.008, 0.05 | 0.60 | 0.546 |
| CME → LTL → DJH | < 0.001 | 0.01 | -0.03, 0.03 | 0.09 | .933 | 0.009 | 0.01 | -0.01, 0.06 | 0.81 | .420 |
| CME → FKA → DJH | 0.04 | 0.02 | 0.001, 0.10 | 1.85 | .065 | -0.02 | 0.02 | -0.06, 0.002 | -1.28 | .199 |
| MCS → LPT → DJH | < 0.001 | 0.001 | -0.01, 0.02 | 0.13 | .901 | 0.03 | 0.02 | -0.01, 0.09 | 1.23 | .217 |
| MCS → LTL → DJH | -0.004 | 0.01 | -0.04, 0.02 | -0.29 | .773 | 0.001 | 0.01 | -0.01, 0.03 | 0.23 | .816 |
| MCS → FKA → DJH | -0.01 | 0.02 | -0.05, 0.02 | -0.36 | .721 | 0.04 | 0.02 | -0.006, 0.10 | 1.54 | .124 |
| CP → LPT → DJH | -0.002 | 0.01 | -0.04, 0.03 | -0.16 | .872 | -0.01 | 0.01 | -0.06, 0.006 | -0.96 | .337 |
| CP → LTL → DJH | 0.01 | 0.01 | -0.003, 0.06 | 1.04 | 0.299 | 0.006 | 0.009 | -0.01, 0.07 | 0.69 | .489 |
| CP → FKA → DJH | -0.01 | 0.02 | -0.07, 0.02 | -0.69 | .490 | 0.008 | 0.01 | -0.008, 0.05 | 0.71 | .475 |
| β, beta-coefficient; BCa CI, bias corrected and accelerated bootstrap confidence interval; CME, core muscle endurance; CP, core power; DJH, drop jump height; FKA, frontal knee angle; LPT, lateral pelvic tilt angle; LTL, lateral trunk lean angle; MCS, maximal core strength; *p*, p-value; *SE*, standard error; *z*, z-value | | | | | | | | | | |

Table 6: Path coefficients of the mediation analyses separated for both the dominant and nondominant legs

|  | **Dominant leg** | | | | | **Non-dominant leg** | | | | |
| --- | --- | --- | --- | --- | --- | --- | --- | --- | --- | --- |
| **Path coefficients** | β | *SE* | 95 % BCa CI | *z* | *p* | β | SE | 95 % BCa CI | *z* | *p* |
| LPT → DJH | -0.02 | 0.14 | -0.32, 0.22 | -0.13 | .900 | -0.18 | 0.15 | -0.46, 0.15 | -1.18 | .237 |
| LTL → DJH | 0.21 | 0.11 | 0.01, 0.45 | 1.89 | .059 | 0.12 | 0.15 | -0.24, 0.37 | 0.75 | .453 |
| FKA → DJH | 0.31 | 0.13 | 0.04, 0.53 | 2.44 | .015 | -0.23 | 0.14 | -0.51, 0.05 | -1.59 | .112 |
| CME → DJH | -0.05 | 0.04 | -0.13, 0.02 | -1.43 | .154 | 0.008 | 0.04 | -0.09, 0.08 | 0.19 | .852 |
| MCS → DJH | 0.21 | 0.03 | 0.15, 0.28 | 6.44 | < .001 | 0.14 | 0.05 | 0.03, 023 | 2.87 | .004 |
| CP → DJH | 0.02 | 0.04 | -0.06, 0.10 | 0.46 | .644 | -0.009 | 0.05 | -0.09, 0.11 | -0.18 | .857 |
| CME → LPT | 0.02 | 0.05 | -0.08, 0.13 | 0.39 | .696 | -0.03 | 0.05 | -0.12, 0.06 | -0.70 | .484 |
| MCS → LPT | -0.01 | 0.06 | -0.13, 0.09 | -0.19 | .852 | -0.14 | 0.05 | -0.25, -0.04 | -2.55 | .011 |
| CP → LPT | 0.11 | 0.06 | 0.004, 0.24 | 1.84 | .067 | 0.07 | 0.05 | -0.02, 0.16 | 1.44 | .150 |
| CME → LTL | 0.004 | 0.05 | -0.10, 0.11 | 0.09 | .933 | 0.08 | 0.06 | -0.04, 0.18 | 1.36 | .173 |
| MCS → LTL | -0.02 | 0.05 | -0.13, 0.10 | -0.30 | .764 | 0.01 | 0.05 | -0.11, 0.10 | 0.25 | .804 |
| CP → LTL | 0.07 | 0.05 | -0.04, 0.02 | 1.27 | .206 | 0.05 | 0.07 | -0.06, 0.19 | 0.78 | .435 |
| CME → FKA | 0.12 | 0.06 | -0.006, 0.21 | 2.16 | .031 | 0.08 | 0.04 | 0.01, 0.17 | 2.13 | .033 |
| MCS → FKA | -0.02 | 0.05 | -0.13, 0.07 | -0.37 | .710 | -0.16 | 0.04 | -0.25, -0.09 | -4.16 | < .001 |
| CP → FKA | -0.04 | 0.07 | -0.15, 0.09 | -0.58 | .565 | -0.04 | 0.05 | -0.14, 0.06 | -0.72 | .472 |
| β, beta-coefficient, Bca CI, bias corrected and accelerated bootstrap confidence interval; CME, core muscle endurance; CP, core power; DJH, drop jump height; FKA, frontal knee angle; LPT, lateral pelvic tilt angle; LTL, lateral trunk lean angle; MCS, maximal core strength; *p*, p-value; *SE*, standard error; *z*, z-value | | | | | | | | | | |
